# Stereological Estimation of Granule Cell Number and Purkinje Cell Volume in the Cerebellum of Noise-Exposed Young Rat

**Published:** 2014-07

**Authors:** Mohammad Hosseini-Sharifabad, Abdoreza Sabahi

**Affiliations:** 1 Department of Biology and Anatomical Sciences, Shahid Sadoughi University of Medical Sciences, Yazd, Iran;; 2 Department of Anatomical Sciences, Isfahan University of Medical Sciences, Isfahan, Iran

**Keywords:** Cerebellum, Noise, Purkinje cell

## Abstract

In spite of the existing reports on behavioural and biochemical changes related to the cerebellum due to noise stress, not much is known about the effect of noise stress on the neuronal changes in the cerebellum. The present study aims at investigating the effects from one week noise exposure on granule cell number and Purkinje cell volume within the neonate rat cerebellum.

15-day-old male Wistar rats were randomly divided into noise exposed (NE) and control groups (n=8 in each group). NE rats exposed to loud noise (100 dB/30 min/3 times per day) during the third postnatal week. One cerebellar half was selected at random for estimating the volume of the cerebellar layers and neuronal quantifications and the other was used for estimating individual somal volume of Purkinje cells. Cavalieri’s principle, physical disector and nucleator methods were employed respectively for unbiased estimation of the volumes of the cerebellar layers, the numerical density of neurons and the individual volume of Purkinje cells.

Results of this study show that noise stress significantly decreases the volume of granule layer together with decreased numerical density and total number of granule cells in the cerebellum. Furthermore, a decrease in somal volume of Purkinje cells was found in NE rats. These results, for the first time, demonstrate an effect of noise stress on the granule cell number and individual volume of Purkinje cells in rat cerebellum.

## Introduction

It is known that the development of the central nervous system (CNS) may be influenced by interactions between the living organism and its environment.^1^ It is reported that loud noise exposure induces disorder involving nervous, endocrine and cardiovascular systems in addition to the direct effect on hearing organs.^2^

Numerous experimental studies concerning the adverse effects of noise stress on the CNS focused on the hippocampus,^3^^-^^5^ but it has rarely been studied in other regions of the CNS. The growing literature demonstrates that noise exposure could induce behavioral changes related to the cerebellum.^6^ It is also well- known that the developmental period of cerebellum extends to the first 3 postnatal weeks in rat^7^ and the long developmental stage of the cerebellum makes it particularly vulnerable to stressful experiences. Furthermore; the oxidative stress in the cerebellum of noise-exposed rats has been reported.^4^^,^^6^ However, not much is known about possible neuronal alterations in the cerebellum caused by noise exposure.

With this background, in continuation of current investigation on the morphometeric changes in discrete rat brain regions due to noise stress, the present study aims at assessing the effects of one week noise exposure on the stereological parameters within the neonate rat cerebellum. 

## Materials and Methods

Pregnant healthy Wistar rats (from animal house of Isfahan Medical School, Iran) were isolated two weeks before delivery. On postnatal day 15, two male offspring weighing 26 g-28 g were taken from each litter and a total of 16 male rats were randomly divided into control and noise exposed groups. Pups remain with the dam during the third postnatal week except for the experiment time at periodic intervals.

Noise-exposed (NE) rats were exposed to a 100 dB un-modulated sinusoidal noise with a frequency of 1100 Hz for 30 minutes, three times per day (10:00, 13:00 and 16:00 h) for one week (postnatal day 15-21). Control rats were moved into a chamber for the same period of experiment time, but without being exposed to noise. 

Animals were housed in plastic breeding cages in an air conditioned room (temperature 23±2ºC) on a 12-hr light/dark cycle (light on at 07.00-19.00 h) with free access to rat chow and tap water throughout the experimental periods. All animal experiments and housing were conducted in accordance with the protocols approved by the research council at medical faculty in Isfahan University of Medical Sciences of Iran.

At the end of the experimental period (day 22 after birth), Plasma corticosterone levels were measured in blood samples drawn from the tail vein. Plasma corticosterone levels were determined by radioimmunoassay using RIA kits (ICN biomedicals). 

Animals were then sacrificed by cardiac perfusion with a phosphate-buffered solution (pH=7.2, M=0.12) of 4% paraformaldehyde (Merk-Germany) and 1% glutaraldehyde (Merk -Germany) under anaesthesia with urethan (Merk -Germany). The cerebellum was separated from the brainstem and was divided at the midsagittal plane. One hemisphere was selected at random for estimating the volume of the cerebellar layers and neuronal quantifications and the other was used for estimating individual somal volume of Purkinje cells.

The cerebellar hemispheres were dehydrated and then infiltrated with glycolmethacrylate (Technovit 7100, Kulzer, Germany). Each plastic-embedded block was exhaustively cut into serial, 1µm semi thin sagittal sections with a rotary microtome ( MICROM International GMBH- Walldorf, Germany). A total of 2900-3300 sections were cut through the entire cerebellar half. From these sections, approximately 12-14 equidistant sections were systematic uniformly random sampled with an initial random start in the first 250 sections, and stained with hematoxylin for quantitative analysis.

The identification of the boundaries of the cerebellar layers was performed using a 4× objective (Olympus, Japan) at a final magnification of 64×. 

The reference volumes “V_(ref)_” of the molecular layer, the granular cell layer and the white matter were estimated using Cavalieri principle^8^ with one section of each consecutive pair selected for the analyses.

The cross sectional areas of the layers of the cerebellum were estimated by point-counting principle with a projection microscope (Zeiss, Germany) using a 4x objective lens at a final magnification of 64×. 

From the obtained sets of semi thin sections, photographs of the same area were taken from the sampled 2 consecutive sections (as above) at a final magnification of 1400×. The physical disector method^9^ was employed to estimate the numerical density (Nv).

The total number of granule neuron is the product of the volume of granular layer multiplied by the numerical density of granule neurons: N=NV×V_(ref)_

Since the sections must be cut with a random orientation for the cell volume estimation, vertical sections^10^ were prepared from other cerebellar half for this purpose. The Purkinje cells to be measured were sampled in disectors on the vertical sections (measurements of approximately 100 cells usually results in an acceptable variation). Photograph of the same area of the Purkinje cell layer were obtained from sets of semi thin sections of the cerebellum and then analysed at final magnification of 1400×.The individual somal volume of Purkinje cells was estimated using the nucleator.^8^

All data were expressed as mean±SD. The statistical significance was evaluated using Student’s t-test by SPSS version 11.5 and P<0.05 was considered to be statistically significant. 

## Results

Findings show that one-week noise exposure significantly reduces the body weight of rats. Furthermore, the brain and cerebellum weights of NE and control rats did not show a significant difference (table 1). Basal plasma corticosterone level was also increased in NE group (20.3±2.13µg/dl) when compared with the control (17.2±1.83µg/dl, P=0.007) group.

**Table 1:** Body, brain and cerebellum weights (g) in control and noise-exposed (NE) rat at postnatal day 22

	**Control** **(n=8)**	**NE** **(n=8)**
Body	40.4±1.84	36.4±1.28*
Brain	1.31±0.11	1.27±0.08 NS
Cerebellum	0.164±0.010	0.155±0.012 NS

Value represent the mean±SD. *P<0.05

Results from the stereological analyses are given in table 2. There was no significant volume change in the molecular layer and the white matter in NE rats in comparison with controls. However, the reduction in the volume of granular layer was statistically significant in NE rats. Data also shows that there is a significant decrease in the numerical density of granule cells in NE compared with controls (1.40±0.064×10^ 6 ^/mm^ 3^ vs 1.53±0.144×10^ 6 ^/mm^ 3 ^, P=0.025). Consequently, a significant decrease (14%) in the total number of granule cell was observed in NE rats. In the NE rats the mean volume of Purkinje cell soma was 15% smaller than those of the control rats (table 2).

**Table 2:** Comparison of stereological parameters in the cerebellum of noise-exposed (NE) and respective controls at postnatal day 22

	**Control ** **(n=8)**	**NE** **(n=8)**	**P value**
Volume (mm3)			
Molecular layer	35.5±2.1	34.6±1.0	0.29
Granular layer	27.0±1.3	25.4±1.2	0.02
White matter	22.1±1.2	21.6±0.7	0.34
Total number (×106)			
Granule cells	41.4±4.5	35.5±2.3	0.005
Somal volume (µm3)			
Purkinje cell	3699±236	3128±398	0.004

All value are expressed as mean±SD

## Discussion

Quantitative results obtained by unbiased stereological techniques showed that exposure of rats to noise (100 dB, 30 min, 3 times per day) during one week from postnatal day 15 to 21 significantly decreases the volume of granular layer together with decreased numerical density of granule cells in the cerebellum. In addition, a decrease in somal volume of purkinje cells was found in NE rats. In agreement with earlier reports,^4^ the plasma concentration of corticosterone also increased due to exposure to noise stress.

Although in this study the mechanisms underlying the reductions in the number of cerebellar granule cells and decrease in the size of perikaryon of purkinje cells were not investigated, however, it is known that glucocorticoids easily cross the blood–brain barrier and acts on CNS. While physiological level of these hormones increases the neuronal communication and promotes the survival of neurons, high plasma levels of glucocorticoids induces neuronal damage.^11^

Previous studies showed that the exposure to high levels of corticosterone during the neonatal period leads to inhibition of the proliferation of neural precursor cells in areas of the brain with postnatal neurogenesis.^12^ During embryonic development of rat cerebellum, granule cells arise between embryonic days 13 and 15, their precursors proliferate in the external granular layer (EGL) and migrate to the internal granular layer over the first postnatal three weeks.^13^ Therefore, in the current study, the duration of noise exposure corresponds to a part of developmental period of cerebellar granule cells. As the final number of granule cells is controlled by cell proliferation and cell death, further experiment is required to determine that a decrease in granule cell number is the result of decreasing cell proliferation or increased cell death induced by elevation of plasma corticosterone due to noise exposure.

Unlike the cerebellar granule cells, purkinje cells are all generated before birth on E13 to E16,^13^ and Purkinje cell dendritic outgrowth and synaptogenesis occur during postnatal three weeks.^14^ Thus, it is expected that noise-induced hypersecretion of corticosterone during the third postnatal week interfere with the maturation of Purkinje cells.

The observed decreases in average somal volumes in purkinje cells of noise exposed rats likely reflect changes at the subcellular level and deserve further investigations.^15^

Current data confirm and extend previous reports that show noise exposure interferes with neuronal proliferation and induces neuronal changes in the CNS of rat. For example, Kim et al. reported that the exposure to the noise during pregnancy decreased neurogenesis in dentate gyrus of the hippocampus.^3^ Saljo et al. also reported that exposure to noise stress induced neuronal death, and apoptosis in granule neurons of the dentate gyrus and the CA1-3 pyramidal neurons in the hippocampus.^5^

## Conclusion

The result of this study indicates that exposure of rats to noise stress during third week of postnatal life significantly decreases both neuronal number in the granule cells and somal volume of purkinje cells in the cerebellum. Further investigations are required to clarify the underlying mechanisms that produce such neuronal changes. 
